# Case management of chiropractic patients with cervical brachialgia: A survey of French chiropractors

**DOI:** 10.1186/2045-709X-19-23

**Published:** 2011-09-24

**Authors:** Olivier Guenoun, Michel Debarle, Coralie Garnesson, Sylvie Proisl, Delphine Ray, Charlotte Leboeuf-Yde

**Affiliations:** 1 Institute Franco-Européen de Chiropratique, 24 blv Paul Vaillant Couturier, F-94200 Ivry sur Seine, France

## Abstract

**Background:**

Not much is known about the French chiropractic profession on, for example, level of consensus on clinical issues.

**Objectives:**

The first objective was to investigate if French chiropractors' management choices appeared reasonable for various neck problem scenarios. The second objective was to investigate if there was agreement between chiropractors on the patient management. The third objective was to see to which degree and at what stages chiropractors would consider to interact with other health-care practitioners, such as physiotherapists, general practitioners and specialists.

**Method:**

A questionnaire was sent to a randomly selected sample of all French chiropractors known to the national chiropractic college. It consisted of an invitation to participate in the study, a brief case description, and drawings of five stages of how a case of neck pain gradually evolves into a brachialgia to end up with a compromised spinal cord. Each stage offered five management choices. Participants were asked at what stages patients would be treated solely by the chiropractor and when patients would be referred out for second opinion or other care without chiropractic treatment, plus an open ended option, resulting in a "five-by-six" table. The percentages of respondents choosing the different management strategies were identified for the different scenarios and the 95% confidence intervals were calculated. There was a pre hoc agreement on when chiropractic care would or would not be suitable. Consensus was arbitrarily defined as "moderate" when 50- 69% of respondents agreed on the same management choice and as "excellent" when 70% or more provided the same answer. It was expected that inter professional contacts would be rare.

**Results:**

The response rate was 53% out of 254 potential participants. The first two uncomplicated cases would generally have been treated by the chiropractors. As the patient worsened, the responses tended towards external assistance and for the most severe case, the majority of respondents would have referred the patient out. There was excellent consensus for the two extreme cases (the most benign and the most severe), moderate consensus for the cases next to these two and least agreement relating to the "middle" case. Inter-professional collaboration was contemplated mainly for the severe case.

**Conclusion:**

The French chiropractors who participated in this study seem to have a similar approach to patients with neck pain that gradually develops into a brachialgia and worsens. However, it is not known if the large group of non-participants in the study would agree with this treatment strategy.

## Background

Chiropractic is a relatively young profession in France. The first North-American educated chiropractors known to have practised in France arrived in 1920[1], and since then chiropractors could be taken to court for illegal practice of medicine. Some chiropractors probably reacted to this threat by claiming that they did not diagnose and treat diseases, similarly to how chiropractors in the US argued their defence when in the same situation in the 1920s and 1930s [2]

Chiropractic was no longer considered to be an illegal profession through a law passed in 2002 [3], and the final law text is in its final stages of completion, at the time of writing this. Compared to several other countries in Europe and elsewhere, this is a late development. Despite this difficulty, the French chiropractic profession has succeeded in establishing its own academic institution with a 6-year undergraduate program.

The number of chiropractors in France is unknown, as there is no registration board, and because not all chiropractors are members of the national chiropractic association. The number having graduated from the French chiropractic college, l'Institut Franco-Européen de Chiropratique (IFEC), between the years of 1984 to 2010 was about 500. It seems reasonable that most of these graduates would have set up their practices in France and that the vast majority of the chiropractors practising in France would be graduates from IFEC. In addition, there are some chiropractors practising in France, who graduated from other colleges elsewhere in the world, particularly before the French chiropractic college existed. In 2011 the Administration at IFEC estimated the number of actively practising chiropractors in France to be approximately 600, in a population of 65 million inhabitants.

It is possible that a small profession with such troubled history has had problems to develop its own "brand" and because the profession is so young it is understandable that it has not had the opportunity to perform research, for example, in order to establish its own demographic profile. Very little is therefore known about the French chiropractors' clinical practice. Further, being relatively isolated from the rest of the chiropractic community, because of the cultural differences and language barrier between England and France, it is possible that French chiropractors practice differently than chiropractors who were educated and/or practise in countries with a strong North-American chiropractic culture. It is therefore relevant to scrutinize the French chiropractic profession from several angles.

Students participating in a research course at IFEC were given the opportunity to perform surveys on the French chiropractic profession. One group decided to study the homogeneity of practitioners in relation to the management of brachial neuralgia emanating from the cervical spine. The cervical spine was an obvious choice for several reasons: Neck pain is a common condition in the general population [4], it is commonly treated by chiropractors [5] and, the neck being a fragile part of the spine, it requires a careful clinical approach in order not to induce injuries of various types. Further, neck pain can be very distressing, and the research team therefore suspected that it might be tempting for the chiropractor to attempt treatment also when this would be unsuitable.

Radiating pain from the neck can be very painful, indeed. An irritation or even inflammation of the nerve root would be the cause of most neck-induced arm pain. This irritation could be either directly or indirectly caused by a bulging or ruptured disc. In the early stages only localized neck pain may be present. As the condition progresses, one would expect to be able to reproduce the arm pain with certain neck movements, by compression of the nerve root within the cervical foramen or by traction of the nerve root. Eventually, if the condition worsens, nerve root signs would become obvious, and if the spinal cord were to be involved, there would also be signs of an upper motor neurone lesion.

The chiropractor is of course expected to diagnose such conditions, and should be able to decide whether manual or other conservative treatment is reasonable or not, and -- in particular -- to be aware of at what stage patients should be referred out for a second opinion or for other type of care. However, to refer out and ask for second opinions may be difficult for practitioners who have never been well accepted within the general health care sector.

There were three objectives of this study:

1. To investigate if French chiropractors' management choices appeared reasonable for various neck-scenarios. It was the opinion of the research team that chiropractic treatment on its own was acceptable in the early stages but more questionable as it became clear that this patient had a more serious condition with a likely disc involvement and totally out of the question when the patient showed signs of spinal cord involvement.

2. To investigate if there was consensus among the chiropractors on the patient management.

3. To ascertain to which degree and at what stages chiropractors would consider interaction with other health-care practitioners, such as physiotherapists, general practitioners and specialists.

## Method

### Study procedure

A team, consisting of three 4th year students and one lecturer in clinical science, was responsible for the design of the questionnaire and the analysis of data. They were assisted by three lecturers in research methodology, of which one was responsible for the logistics of the mailing out and reception of questionnaires.

A cross-sectional study was carried out between the 5^th ^of October and 18^th ^of November in 2009. From a list of 634 chiropractors known to practice in France at that time, a random selection was extracted consisting of 254 individuals. The remaining chiropractors were asked to participate in other surveys. The list of the targeted study population had been obtained through the membership list of the French chiropractors' association, telephone directories and the internet.

Each chiropractor in the randomly selected study sample received an envelope containing a letter of information, an addressed and pre-stamped envelope, and a questionnaire. A dead-line for answering was set and participation was voluntary and anonymous. After 10 days, a second letter was sent out with a request to respond to the questionnaire, if this had not already been done.

### The Questionnaire

The questionnaire consisted of the following general information: "A 28-year old man, tennis player by profession, consults you for a right-sided intense neck pain without any radiating pain. You note an antalgic position of the head, no other musculoskeletical signs (no torticollis), no other health problems in particular, normal x-rays for his age, and no signs of alert (red flags)."

Five scenarios were thereafter presented, under the question. "In each of the cases described below, what would you do?" Each scenario consisted of a simple drawing of a mannikin showing the posterior aspect of the upper torso, arms and head, and a brief explanation of the case at the side of this mannikin. The five scenarios were as follows:

1. The mannikin was marked with a red cross in the lower cervical spine to indicate the area of pain. The text said: "Physical examination: very tense cervical musculature, no neuro-vascular problems, right C5-6 painful on palpation, pain 7/10 on a visual analogue scale".

2. The mannikin was marked with a red cross in the same spot and, in addition, an uninterrupted area of pain was drawn from the cross in the lower neck to the tip of the shoulder. The text read: "Five days later the patient comes back to you: Same clinical signs but the pain now radiates into the right shoulder".

3. The cross remained in the third drawing but the area of pain now descended all the way to the elbow. The following written information was provided: "Four days later: An MRI reveals a postero-lateral discal hernia at C5-6 which affects the C6 nerve root. There is now a neurological sign: C6 reflex diminished (1+), normal myotomes and dermatomes."

4. The pain drawing now reveals a pain from the base of the neck down the posterolateral side of the arm including the whole thumb. The accompanying text said: "Another 4 days later: The neck pain is gone. The neurological signs are obvious: C6 reflex absent (0), the C6 myotome diminished (2), C6 dermatome disturbed (reduced sensitivity)."

5. The pain drawing is the same and the accompanying text said: "Ten days later: The symptoms and signs are the same as last time but in addition the following is noted: the right leg shows hyperreflexia (3+), a positive sign of Babinski on the right and slight hypoaesthesia of the right leg."

There was a choice of 6 answers, for each of the five scenarios. These consisted of five defined management choices and one open ended question. These options were: 1. I treat the patient on my own. 2. I treat the patient with the assistance of some paramedics. 3. I treat the patient with the assistance of a general practitioner. 4. I treat the patient whilst asking the opinion of a specialist. 5. I do not treat the patient but refer him out. 6. Other, please explain on the last page.

The following three sentences were provided as footnotes: 1. In this study, kinesitherapists, ergotherapists and physiotherapists are considered to be paramedics. 2. Only neurologists, rheumatologists and surgeons are considered to be specialists. 3. If you decide to refer to some other health care professional, not mentioned here, please tick "other" and explain further on the last page.

On the last page, space was made available to write comments for each of the five scenarios, and the participants were thanked for their assistance.

### Ethical considerations

Participation in the study was voluntary, anonymous, and no additional demographic data were collected, making it impossible in any way to identify the participants. The questions were innocuous and unlikely to cause any personal distress. There was no involvement of patients and no experiment was carried out. Therefore, the Ethics Committee at IFEC did not wish to review the research project and no ethics acceptance of the study was necessary.

### Expectations

As a minimum, we expected that all chiropractors would prefer to treat the patient described in the first scenario (uncomplicated local neck pain) without assistance or referral and that all participants would refrain from treating the patient described in the last scenario (spinal cord signs), and instead refer out.

We also expected that the choice of treatment option would shift to the right side of the questionnaire as the condition worsened, i.e. that the chiropractors would increasingly require assistance from other health professionals, when the scenario indicated other than uncomplicated neck pain or relatively benign radiating pain.

We expected there to be high consensus in the two extreme scenarios (most benign and most serious) and moderately high consensus on the three strategies in between.

We also suspected that there would be only modest signs of collaboration with other health care practitioners.

### Analysis of Data

Analysis of data was made by counting the types of response for each question. However, sometimes comments written under "other" could be placed in one of the pre-worded options. The option "other" was only retained when this box had been ticked but no other information was provided or when that information could not be used to fit the answer into any of the other options. When chiropractors provided several answers to one question, this was coded as "other".

The most frequently selected choice in each category has been highlighted in Table 1. Estimates reported in the text are accompanied by their 95% confidence intervals (CI) in order to provide an indicator of measurement error or uncertainty of estimates. Consensus was arbitrarily considered to be "moderate" when at least 50% of clinicians agreed on the treatment option and "excellent" if at least 70% agreed on a treatment strategy.

**Table 1:** Consensus (reported as percentages) on the choice of treatment strategy amongst 135 French chiropractors, regarding five different clinical scenarios with a choice of five clinical management options

Case scenarios	Treat self	Treat with paramedical assistance	Treat with GP assistance	Treat with specialist assistance	Do not treat but refer out	Other	No response or several answers
1.Local neck pain	87	2	6	<1	2	2	0

2.Neck pain and pain radiates to tip of shoulder	57	4	24	4	4	4	2

3.Neck pain and pain radiates to elbow, reduced C6 reflex	27	2	14	35	10	8	3

4.No neck pain. Arm pain radiates to include thumb, neurological signs C6	6	0	6	26	50	7	5

5. No neck pain. Arm pain and upper motor neurone lesion findings in lower limb.	4	0	0	12	74	3	7

The estimate with the highest percentage agreement is highlighted for each scenario.

## Results

Of the 254 questionnaires, 139 were returned, of which 4 were empty, giving a response rate of 135/254 (53%). In order to protect the identity of the chiropractors, no additional demographic information had been requested, thus making it impossible to perform a non-responder analysis.

As can be seen in Table 1, which provides all estimates discussed in this report, in all five scenarios, less than 10% of the respondents had given "other" suggestions to treatment options than those provided in the questionnaire. Between 0 and 7% of the five scenarios were not provided with a response.

### Responsible practice pattern

In general, there was a reasonable pattern of responses, with the vast majority (87%; 95% CI 80-94) claiming that they would "treat self" the local neck pain and 57% (49-65) would do so also when the pain radiated to the tip of the shoulder.

However, when the pain radiated to the elbow with a reduced C6 reflex, 49% (CI 41-57) would treat with a generalist or specialist assistance. When obvious neurological signs appeared, 76% (69-83) would either refer out or treat with specialist assistance, and 74% (67-81) would refer out the patient with obvious signs of an upper motor neurone lesion.

There were very few respondents who showed signs of obviously inappropriate treatment behaviour. There were 2% (0-4) who would not treat but refer out the patient described in scenario 1 whereas 4% (1-7) would treat without referral or co-management the patient described in scenario 5.

### Consensus between clinicians

There was excellent consensus for the first and the last two scenarios (the most benign and the most severe), moderate consensus for the two scenarios next to these but only 35% (27-43) consensus for the "middle" case (neck pain radiating to elbow, reduced C6 reflex).

### Intention to collaborate with other health practitioners

It was very rare (0-4%) to treat with paramedical assistance. However, assistance from a general practitioner was suggested by 24% (17-31) already when the arm pain radiates to the tip of the shoulder and by 14% (8-20) when the pain radiates to the elbow with a reduced C6 reflex, but none would consider the GP for a patient with an upper motor neurone lesion. However, assistance from a specialist would be called upon as the case became more severe; 35% (27-43) would call upon help when the reflex diminished, 76% (69-83) would either seek assistance from a specialist or refer out (most likely to a specialist) when there were obvious neurological signs, and 74% (67-81) would refer out when there were upper motor neurone findings.

## Discussion

### Summary of findings

Patients who consult any of the chiropractors who participated in this study can expect a relatively similar overall approach to neck pain and radiating arm pain and this approach appears to be largely acceptable.

### Methodological considerations

However, the response rate was low, only 53% of questionnaires were returned for the analysis, and it is not known if the 47% non-responders would have a different attitude to the treatment approach for this type of patient. Therefore, it would have been an advantage, if it were possible to make a comparison of responders and non-responders, at least on college of graduation, age, and area of work, to see, if the two groups resembled each other. However, due to privacy issues, it was decided not to collect any demographic data on the participants, as this could have been used to identify some of the clinicians. It was thought that this could help increase the participation rate, which we had anticipated to be rather low. It is not known, if this strategy worked or not.

Questionnaires are commonly tested in pilot studies. However, the present questionnaire that was designed by the research team was considered so basic and simple that no pilot study was conducted to test its user friendliness. Members of the academic staff did, however, proof-read the document for logic and absence of errors. Because of the low percentages of "no response" throughout the questionnaire we assumed that it was easy to understand and respond to.

It is our experience that clinicians sometimes claim it impossible to answer clinical questions in simple terms. There is therefore a need, in questionnaires of this type, to include the possibility to provide "other" answers. The percentages of "other" answers ranged between 2% and 8%, which is somewhat higher than in a previous similar Swedish study on management choices for low back pain, where this type of open response was selected by about 2% of the chiropractors [6]. However, the Swedish chiropractors were used to responding to questionnaires, since they had participated in a number of studies already, which might explain this difference.

### Consensus

The consensus between clinicians appears fairly high although only excellent in the two extreme cases. In comparison, estimates as high as 74% and 87% were not found in the previous survey of Swedish chiropractors, who were asked about their management strategies for a number of low back pain scenarios [6]. One reason for this could be that it is easier to agree on the management of neck pain than that of low back pain. Another possible reason could be that there were more scenarios and more treatment options to choose between for the scenarios used in the Swedish study.

### Clinical acceptability and collaboration with other health care professionals

Consensus is, of course, a good thing, as it unites a profession and provides it with a "brand" that is recognizable by the public. However, agreement between professionals to act irresponsibly is not desirable. The results of this study indicate that the responders of this study, on the whole, have a responsible approach to this type of neck condition.

We had expected that only a few chiropractors would use the assistance of other professional groups (paramedics, general practitioners or medical specialists), reflecting the long-term professional isolation that chiropractors have been subjected to in France. When there is a risk of prosecution for the illegal practice of medicine, the distance between the chiropractor's office and that of other practitioners can be very long. There is also no tradition in France of chiropractors working together with medical practitioners and physiotherapists in clinical teams. Nevertheless, collaboration with other health-care professionals was, at least, contemplated for the more serious situations. This does not imply that such contacts are very frequent, as such conditions would be relatively rare in primary care, and we did not study the frequency of inter-professional contacts per se.

Of concern, in the case of the obvious contra-indication to chiropractic care (signs of a spinal cord lesion, suitable for immediate referral to neurosurgeon or orthopaedic surgeon), there were 4% who would select continued care and 12% who would continue treatment albeit in collaboration with some specialist. On the other hand, it is curious that for the simplest scenario, 2% would refer the patient out of the clinic and about 7% would treat this patient in conjunction with a general practitioner or a specialist.

## Conclusion

The participants in this survey agreed largely on the choice of treatment strategies for a patient with neck pain, which was described to develop increasingly severe signs and symptoms, and, in general, their strategic choices appear to be sound and safe. Collaboration with other health-care practitioners would probably mostly be contemplated for contra-indications and possible contra-indications to spinal manipulative therapy.

## Competing interests

The authors declare that they have no competing interests.

## Authors' contributions

This article is based on a research project conducted as a part requirement for the chiropractic degree at the Institut Franco-Européen de Chiropratique for CG, SP and DR. All authors participated in the design of the study. On-site supervision was performed by OG and MD. CLY was responsible for the overall supervision and the final draft. All authors read and approved of the final version.
